# Increased Digital Media Use in Preschool Children: Exploring the Links with Parental Stress and Their Problematic Media Use

**DOI:** 10.3390/children10121921

**Published:** 2023-12-13

**Authors:** Elena Kattein, Hannah Schmidt, Stefanie Witt, Hannah Lea Jörren, Ingo Menrath, Hans-Jürgen Rumpf, Lutz Wartberg, Silke Pawils

**Affiliations:** 1 Department of Psychiatry and Psychotherapy, University of Lübeck, 23562 Lübeck, Germany; 2 Department of Paediatrics and Adolescent Medicine, University of Lübeck, 23562 Lübeck, Germany; 3 Institute of Medical Psychology, University Medical Center Hamburg-Eppendorf, 20251 Hamburg, Germany; 4 Department of Psychology, Faculty of Human Sciences, MSH Medical School Hamburg, 20457 Hamburg, Germany

**Keywords:** digital media use, problematic internet use, internet addiction, Internet Gaming Disorder, gaming disorder, children’s media use, parental media use, parental stress, prevention, preschool children

## Abstract

Background: Intense or problematic media use behavior of parents could serve as a role model for their children’s media use behavior. So far, knowledge is scarce about increased children’s media use (ICMU) and its association with parental stress (PS) and problematic parental media use (PPMU). Methods: ICMU was examined using a modified set of the DSM-5 criteria for Internet Gaming Disorder. PS was assessed via the widely used Parenting Stress Index, and PPMU was assessed using the Short Compulsive Internet Use Scale. A multiple linear regression analysis was conducted to evaluate the links between ICMU, PS, and PPMU. A mediation analysis was performed to examine if PPMU mediated the relationship between PS and ICMU. Results: In sum, 809 parents (*M* = 36.89 years; *SD* = 4.87; 81.4% female) of preschool children (average age: 44.75 months, *SD* = 13.68) participated in the study. ICMU was statistically significantly related to higher age of the parent, greater PPMU, and higher PS. Furthermore, we found that the association between PS and ICMU was partially mediated by PPMU. Conclusions: The results indicate that more pronounced PS and PPMU are associated with ICMU, highlighting the necessity of considering these parental variables when developing early prevention strategies for preschool-age children.

## 1. Introduction

From birth onwards, children grow up in a media-influenced environment with both traditional and new forms of digital media. Typically, traditional media (also known as broadcast media) include the “passive” use of devices, such as television or radio, with content that is usually created externally by an established producer [1]. In contrast, digital media focus on users’ own interactive and social engagement. Users can both consume and actively create content, for example, via social media applications (apps) [1]. On average, children in Germany have their first exposure to digital devices already at the age of twelve months [2]. Even preschool children, who have not yet developed reading and spelling skills, can use and navigate through digital media guided by symbols [3].

### 1.1. Benefits and Risk of Children’s Media Use

Digital media, when approached mindfully and under parental supervision, can indeed offer several benefits for children, for example, individualized learning opportunities and the development of various skills via educational apps [4,5]. Additionally, digital media may enhance children’s creativity by enabling them to express themselves through digital art music and storytelling [6]. Digital media may also provide tools and apps that support children with special needs (e.g., with chronic conditions) by offering them resources for communication, learning, and skills development [7].

Despite these benefits, there are also risks of prolonged and unmonitored digital media use, in particular for young children. In previous studies, it was already reported that increased digital media use among children is associated with a sedentary lifestyle, for example, physical inactivity and unhealthy nutrition behavior [8,9]. Increased use of digital media, especially before bedtime, can disrupt sleep patterns and lead to insufficient sleep quality [10]. Furthermore, unrestricted access to age-inappropriate content can lead to exposure to violence, explicit material, or other content that may be harmful for children [11]. Most importantly, increased digital media use in childhood may hinder the development of communication skills and the building of real-life relationships with family and peers, and may impair other crucial aspects of life, such as hobbies and sports [12]. Some children, especially those with additional vulnerabilities or challenging family situations, may face an increased risk of developing addiction-like media use behaviors in their youth. Therefore, it is an important developmental task for children to learn how to use digital media responsibly, and it is essential to explore factors that are associated with their digital media use habits.

### 1.2. Parental Impact on Their Children’s Media Use

Family aspects, especially in the first years of a child’s life, have a considerable influence on development and behavior. Similarly, it can be assumed that the digital media use of children is also highly influenced by their parents (which has already been shown empirically for adolescence, e.g., [13]). Parents play a pivotal role in establishing rules for the time and content of digital media [14]. However, several parents experience high levels of stress and mental load in daily life by balancing job, childcare, and household and often resort to providing digital devices to calm or distract their children. Already in preschool age, pilot studies have found a negative link between high parental stress and problems in limiting the digital media use of three- to five-year-old children (e.g., [15]). In the short term, children’s digital media use may reduce oppositional behaviors. However, in the long term, this can negatively reinforce a lack of parental control, making it challenging for parents in the long term to restrict access to digital devices as desired.

In addition to parental stress, it can be assumed that parents’ own use patterns may also play a role in their children’s digital media use since young children often emulate parental daily habits. Although there are limited empirical studies on this connection at a young age, it is reasonable to assume that children are likely to adopt similarly increased media use habits as their parents. While the media use of children aged zero to three has been infrequently explored, further research considering family factors (such as parental stress and their problematic media use) is important for a better understanding of increased media use among children.

### 1.3. How to Measure “Problematic Media Use” at Preschool Age?

Defining and measuring “problematic media use” in early childhood is a methodological challenge. In adolescence and adulthood, the problematic use of activities on the Internet often refers to specific diagnostic criteria that go along with functional impairments in daily life (see the end of this paragraph for further definitions). Although some parents even use the term “addiction” to describe their children’s digital media use behavior, many researchers refrain from using this concept for this age group and, instead, define it as problematic media use. The rationale for this is that preschool children’s media use takes place in the context of their family, which means that parents play a crucial role in setting limits and granting access to digital devices (e.g., [14]).

To date, most studies analyzing children’s media use have exclusively focused on the time spent with media by a child. The German Federal Centre for Health Education (BZgA) and other experts recommend that children aged zero to three years should completely avoid screen media to prevent negative impacts on their mental and physical health [16]. At the preschool age between 3 and 6 years, it is recommended to use digital media for a maximum of 30 min daily [16]. For primary school-aged children between 6 and 10 years, the BZgA recommends using digital media 45 to 60 min per day [16].

In daily life, however, it is often challenging to strictly adhere to these guidelines offered by the BZgA. The COVID-19 pandemic led to increased media use habits, as reported by 14% of parents mentioning their preschool children spending more time online and 33% playing with computers or smartphones more frequently [17]. However, time alone may not be a reliable predictor of problematic media use by children. Similar to adolescents and adults, it seems of importance to also observe changes in specific behavior, such as aggression, irritability, or mood swings, which may be linked to media use habits and the experience of a person in connection with specific devices.

Even for adolescents and adults, the conceptualization of the phenomenon of “Internet Use Disorder” is currently controversially discussed in research. One exception is the diagnosis of Internet Gaming Disorder (IGD), which was incorporated in 2013 in the fifth version of the Diagnostic and Statistical Manual of Mental Disorders (DSM-5) due to the evidence in the scientific literature [18]. Additionally, Gaming Disorder (GD) has been included since 2018 in the International Classification of Diseases-11 (ICD-11; [19]). The DSM-5 is often used in international research. IGD is defined as the problematic use of video games for both online and offline gaming [18]. An individual is considered to have IGD if she or he meets at least five of the nine criteria in the past 12 months. According to the DSM-5, these criteria are (I) strong mental and emotional involvement, (II) withdrawal, (III) tolerance, (IV) unsuccessful attempts or persistent desire to limit or completely give up gaming, (V) loss of interest in other activities due to gaming, (VI) excessive gaming despite negative consequences, (VII) deceiving others about the extent of gaming, (VIII) use of gaming to escape negative moods, and (IX) endangerment or loss of relationships, a job, or education-related or professional opportunities due to gaming [18]. Up to now, the IGD criteria are only applied to adolescents and adults. To develop tailored preventive approaches, research is now needed to examine if the generalized IGD criteria are also applicable for younger children.

### 1.4. Aim of the Present Study

In the present study, associations between increased children’s media use (dependent variable) as well as parental stress and problematic parental media use (explanatory variables) were surveyed. The following hypotheses were tested:

(a) Increased children’s media use is related to parental stress and problematic parental media use. These relationships persist even when considering sociodemographic variables (gender and age of the parents as well as perceived socioeconomic status).
(b) Problematic parental media use mediates the association between parental stress and increased children’s media use.

## 2. Methods

### 2.1. Participants and Procedure

**Figure 1:**
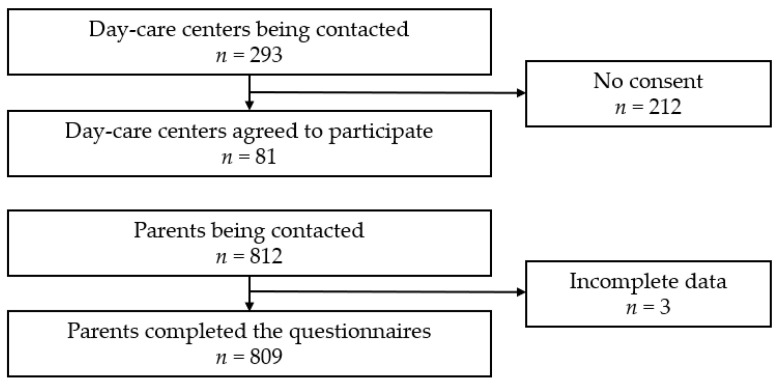
Flow chart.

The recruitment of parents was conducted in day-care centers in two federal states in Germany (Hamburg and Schleswig-Holstein) between January and October 2021. The inclusion of data from two German federal states, covering both rural and urban day-care centers, was intended to improve the generalizability of the findings. The completed questionnaires were received until 31 January 2022. In total, 293 day-care centers were contacted and informed about the study by mail. Of those, 81 agreed to participate in the survey, and they subsequently received quarterly questionnaire packets and consent forms. These were distributed by the educators to parents of preschool children. The only inclusion criteria to participate in the survey were to be a parent of a preschool child and to have sufficient language skills in German to answer the questionnaire. In total, 812 participants were screened. Due to missing data, 809 questionnaires could be finally included in the statistical analyses. The study design and procedures are shown in Figure 1.

### 2.2. Measures

#### 2.2.1. Sociodemographic Data

The sociodemographic data collected included gender and age (in years) of the parents as well as gender and age (in months) of the children. The parents’ (self-)perceived socioeconomic status (SES) was assessed with the adoption of the McArthur Scale [20]: “Consider a hypothetical scale with 10 rungs representing socioeconomic status in Germany. Rung 10 signifies individuals with the highest income, the most advanced education, and the most prestigious employment, while rung 1 represents those with the lowest income, minimal education, and precarious or no employment. Reflecting on your own circumstances, where would you position yourself on this scale (1–10)?”. All sociodemographic data were recorded using single items.

#### 2.2.2. Increased Children’s Media Use

Increased children’s media use was measured via parental rating of four adapted items based on the DSM-5 criteria for Internet Gaming Disorder [18]. The four questions were changed in their wording to “digital media use” and were adapted to preschool child behavior. Three of the four item formulations were previously published as part of the parental version of the Young Diagnostic Questionnaire (PYDQ, [21]), an established instrument to capture parental assessment of their children’s problematic Internet use (e.g., [22]). The four questions measure the DSM-5 criteria of tolerance, craving, emotion regulation, and conflicts, with a five-level response format (0 = “never” to 4 = “very often”). An example item is “Does your child feel restless, moody, depressed or irritable when you try to reduce digital media use?”. The reliability (Cronbach’s alpha) of the instrument in the surveyed sample was 0.86. A higher sum value of the four items indicates a stronger increase in children’s media use.

#### 2.2.3. Parental Stress

Parental stress was recorded via the German version (“Eltern-Belastungs-Inventar”, EBI; [23]) of the widely used Parenting Stress Index (PSI). Parental stress is defined as a comprehensive term for stress-inducing situations that stem from the role of being a parent [23]. Central to this definition is the subjective perception of parents regarding the availability of personal resources relative to the perceived demands associated with parenthood [23]. The EBI questionnaire comprises 48 items (an example item is “I feel trapped by my responsibilities as a parent”), with a five-level response format (1 = “strongly disagree” to 5 = “strongly agree”). It evaluates both overall parental stress as well as two distinct domains: the parent domain and the child domain. The child domain encompasses aspects of parental stress related to one’s child and includes the five subscales: “hyperactivity/distractibility”, “mood”, “acceptability”, “demand”, and “adaptability”. For example, one of the items is “My child is much more active than other children” [23]. The parent domain incorporates seven subscales, including “parental attachment”, “social isolation”, “parental competence”, “depression”, “health”, “personal restrictions”, and “partner relationships”. An example question from this domain is “Since I had my child, I have been sick more often” [23]. The reliability coefficient of the EBI sum score in the sample examined was 0.95. A higher EBI sum score indicates more pronounced parental stress [23].

#### 2.2.4. Problematic Parental Media Use

Problematic parental media use was assessed using a short form of the Compulsive Internet Use Scale (CIUS; [24], whose German version had shown very good psychometric properties in representative samples, e.g., [25]). This brief screening instrument (abbreviation: Short CIUS, [26]) consists of five items (an example item is “How often do you neglect your everyday responsibilities because you prefer to go on the Internet?”) also with a five-level response format (0 = “never” to 4 = “very often”). The reliability coefficient of the Short CIUS in the sample investigated was 0.78. A higher Short CIUS sum score indicates a stronger pronounced problematic media use of the parents.

#### 2.2.5. Statistical Analysis

Most of the data analyses were performed using SPSS Statistics version 27.0 (IBM, 2020, New York, NY, USA). The significance level was set to *p* < 0.05. We calculated the means (M), standard deviations (SD), and frequencies and performed reliability analyses. To explore the associations between increased children’s media use (dependent variable), parental stress, and problematic parental media use while adjusting for gender and age of the parents as well as perceived socioeconomic status (explanatory variables), a multiple linear regression analysis was performed (test of Hypothesis 1). Furthermore, a mediation analysis (according to MacKinnon [27]) was performed to examine if problematic parental media use mediated the relation between increased children’s media use and parental stress (test of Hypothesis 2). This mediation analysis was conducted using the statistical software Mplus version 8.10 [28]).

#### 2.2.6. Ethics

The study procedure was carried out in accordance with the Declaration of Helsinki. A comprehensive data protection protocol ensured the pseudonymity and secure handling of the participants’ data. This study was reviewed by the Psychological Ethics Committee at the Centre for Psychosocial Medicine and approved on the 7 July 2020 (file number: LPEK-0117), and reviewed by the Ethics Committee of the University of Lübeck and approved on 8 August 2020 (file number: 20–310). All participants were informed about the aim of the study. Informed consent was obtained from all participants in advance, and participation in this study was voluntary.

## 3. Results

### 3.1. Sample Characteristics

The description of the sample (*N* = 809 parents) of the present study is presented in Table 1.

### 3.2. Association of Children’s Media Use, Parental Variables, and Sociodemographic Variables

Increased children’s media use was associated with higher age of the parent, higher parental stress, and more pronounced problematic parental media use (see Table 2). About one sixth of the variance in increased children’s media use (Corrected *R*^2^ = 0.16) was explained by the multivariable regression model.

### 3.3. Mediation Analysis

**Figure 2:**
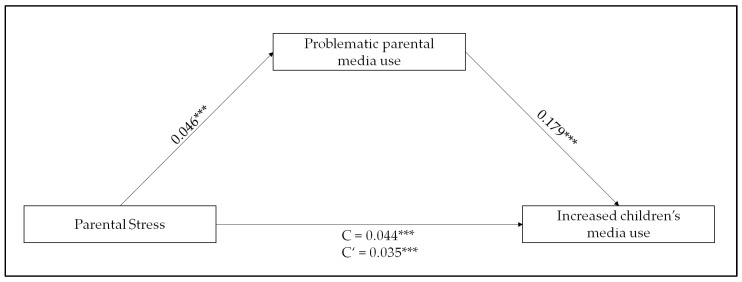
Parental stress and increased children’s media use mediated by problematic parental media use. Note: *** *p* < 0.001.

Parental stress had an association with increased children’s media use (total effect or C in Figure 2 was B = 0.044, 95% Confidence Interval (CI): 0.035 to 0.052, *p* < 0.001). Parental stress showed a significant association (B = 0.046, 95% CI: 0.038 to 0.054, *p* < 0.001) with the mediator (problematic parental media use), which, in turn, displayed a relationship with increased children’s media use (B = 0.179, 95% CI: 0.097 to 0.262, *p* < 0.001). The association between parental stress and increased children’s media use was partially mediated by problematic parental media use (indicated by a total indirect effect of 0.08, 95% CI: 0.004 to 0.012, *p* < 0.001; the direct effect or C’ was B = 0.035, 95% CI: 0.026 to 0.044, *p* < 0.001).

## 4. Discussion

The present study aimed to investigate potential associations between increased children’s media use with parental stress and problematic parental media use. In line with our first hypothesis, we observed an association between parental stress and increased children’s media use. This result fits well with published studies [29,30,31,32,33,34]. For example, a prior study concerning IGD in children and adolescents reported links with elevated levels of parental depression and anxiety, which may, of course, also contribute to more pronounced parental stress, and vice versa [30]. The association between parental stress and children’s increased media use can be attributed to several interconnected factors. One possible explanation is that parents experiencing high stress levels may turn to digital media as a coping mechanism. The use of smartphones or tablets can serve as a distraction, offering a (temporary) escape from stressors and challenges in the immediate environment or to distract from their children, especially if their children show a challenging behavior [32]. Children, on the other hand, often model their behavior in line with their parents. If parents are frequently using digital media to cope with stress, children may observe and follow the parental role model [33].

A second explanation might be that stressed parents might inadvertently use digital media as a distraction while interacting with their children. This distraction can lead to decreased engagement with their children, potentially resulting in more lenient attitudes towards their children’s media use. Stressed parents may find it challenging to be fully present and engaged with their children. Due to their own fatigue or a desire to avoid conflicts with their children, parents might allow their children more unrestricted access to digital media for entertainment, providing a temporary reprieve for the parents themselves. Previous studies found associations between increased maternal stress and increased screen time in toddlers [29], a finding that also fits well with the results of the present study. It was reported that elevated stress levels in mothers can promote fewer restrictions on screen use for young children [15]. Given that more than four out of five parents in the present study were female, this could also be an explanation for increased children’s media use. Moreover, high levels of stress in parents may lead to feelings of guilt about not spending enough quality time with their children. To alleviate guilt or as a permissive response, parents may allow more extensive use of digital media by their children.

In line with our second hypothesis, we found that problematic parental media use mediated the association between parental stress and increased children’s media use. The obtained association between problematic parental media use and increased children’s media use is in line with the findings of previous studies [35,36,37,38,39,40]. To date, most published studies have mainly focused on older children. In this study, the link between increased children’s media use and problematic parental media use is now also shown in a considerably younger population. Parents using digital devices in the presence of their children could serve as a role model for preschool children’s media use. It could be assumed that the more actively parents interact with the Internet, the more actively the children themselves use internet-based devices, regardless of the Internet application. Despite these empirical findings, in everyday life, parents often tend to underestimate their influence on their children through their own digital media use [39]. It is, therefore, important that parents set an example of appropriate use of digital devices and, if necessary, encourage their children to reflect critically on their use of digital media [40,41]. Accordingly, parents and their media use could also be an important starting point in prevention to forestall increased media use of preschool children.

Our findings indicate that parents who demonstrate responsible and mindful media use themselves might serve as positive role models for their children. They might influence them to adopt similar habits by educating them about age-appropriate content. Keeping an eye on the media content children consume and supervising their online activities allows parents to ensure appropriateness and intervene if necessary [38]. Additionally, designating specific areas or times without digital media helps establish boundaries and promotes face-to-face interactions within the family, and encouraging a balance between screen time and other activities, such as outdoor play, reading, or creative pursuits, helps shape a well-rounded approach to media use [38]. However, according to our findings, it also appears relevant that parents themselveshave sufficient coping strategies for stress management to be able to implement such media rules in their family system. In general, the mental well-being of parents (including their stress level) is a crucial factor influencing their children’s development, particularly during the first years when parents have significant influence on their toddlers. In this case, pediatricians, child psychologists, educational counseling centers, youth welfare office, or other professionals in the pediatric care system might be a low-threshold resource for families that would like to obtain advice on how to develop individual family rules in terms of media use and support their efforts to create boundaries in their households [38]. Our findings underscore the importance of considering family dynamics and parental well-being when addressing children’s media habits. Interventions and support for parents to manage stress in healthier ways can potentially impact children’s media use positively.

The present investigation has several limitations. The sample consists mainly of mothers, many of them with perceived medium-to-high SES. This poses a limitation to the generalizability of these findings, especially since parents in low-SES families and single parents often experience even more increased levels of parental stress. Furthermore, the assessment of increased children’s media use is reliant on parental reports, which might introduce potential biases due to social desirability. Additionally, the findings might be influenced by the context of the COVID-19 pandemic, which promoted a general increase in parental stress. Parents might be more prone to engage their children with digital media due to the temporary closure of daycare centers during the COVID-19 pandemic. Moreover, parents might have become more aware of potentially problematic behaviors in their children during the pandemic due to increased health concerns. Another significant limitation arises from the cross-sectional design of the study, which precludes the establishment of any causal relationships. In this study, we consciously focused solely on parental variables associated with increased children’s media use. This choice was purposefully made because parental factors have a transdiagnostic and significant impact on the well-being of their children, particularly in this very young age group of preschoolers. However, to develop age-appropriate prevention strategies for children’s increased media use, it is essential to expand the scope and to also explore other potentially relevant parental (e.g., personality traits) and child-related variables. One potential variable for future consideration is children’s ability for emotional regulation. For example, research involving Chinese preschoolers aged 42 to 72 months revealed a positive link between their digital media use and the risk of social-emotional developmental delays [42]. Moreover, parental media usage was recognized as a mediating factor in this association.

One of this study’s strengths is its utilization of a selection of diagnostic criteria from the DSM-5 for assessing increased children’s media use. This is particularly noteworthy, given that these criteria are primarily designed for youth and adults. Furthermore, the present study focuses on a very young sample of preschool children. This age group has been, so far, rarely studied, and this study allows the integration of the observed associations into the development of potential primary prevention strategies.

## 5. Conclusions

To date, children grow up with early digital media use experiences. Despite several benefits of digital media use, families should be aware of the potential risk for development of increased or even problematic children’s media use. The aim of this study was to investigate possible links between parental variables and increased children’s media use, measured not only based on the time spent with media but also based on a set of adapted DSM-5 IGD criteria. Our findings support our hypotheses of the links between parental stress, problematic parental media use, and increased children’s media use. Hence, parents should be aware of and understanding their own role in setting appropriate media use for their children. It seems of importance to watch and discuss media together with children, in particular preschool-age children. This shared experience enables parents to guide their children’s understanding of media content, answer questions, and address any concerns. Future research could involve identifying further parental risk factors (e.g., media educational behavior or personality traits of parents) and children’s risk factors (e.g., deficient emotion regulation strategies) to enhance potential prevention programs for children’s problematic media use. This may include educating families on effective stress management strategies in the context of digital media use. Additionally, longitudinal studies could shed light on whether parental stress leads to increased children’s media use, or vice versa.

## Figures and Tables

**Table 1:** Sample characteristics.

	Total Sample, % or *M* (*SD*)
Age of the parent ^1^	36.89 (4.87)
Gender of the parent	
Male	12.5%
Female	81.4%
Both ^2^	5.9%
Age of the child ^3^	44.75 (13.68)
Gender of the child	
Male	52.9%
Female	47.1%
Perceived socioeconomic status	6.89 (1.57)
Increased children’s media use	5.28 (3.63)
Parental stress	117.95 (30.27)
Problematic parental media use	4.85 (3.39)

Notes: ^1^ = In years. ^2^ = Both parents had filled out the questionnaire together. ^3^ = In months.

**Table 2:** Multivariable regression model on associations between increased children’s media use, parental stress, problematic parental media use, and sociodemographic variables.

Variables	*β*	*p*
Age of the parent	0.10	0.010
Gender of the parent	0.03	0.389
Perceived socioeconomic status	−0.06	0.134
Parental stress	0.30	<0.001
Problematic parental media use	0.17	<0.001

## Data Availability

The research data can be requested from the corresponding author. The data are not publicly available, as the authors want to carry out further analyses and publish them before the data can be made available to the public.
